# Supplementary material for: Exosomal circEZH2_005, an intestinal injury biomarker, alleviates intestinal ischemia/reperfusion injury by mediating Gprc5a signaling
Source: Nat Commun. 2023 Sep 6;14:5437. doi: 10.1038/s41467-023-41147-3 (PMC10482849; doi:10.1038/s41467-023-41147-3)
Supplement: Supplementary file 1 — Supplementary Information [file 41467_2023_41147_MOESM1_ESM.pdf]

Supplementary Information for

Exosomal circEZH2_005, an intestinal injury biomarker, alleviates intestinal ischemia/reperfusion injury by mediating Gprc5a signaling

Wenjuan Zhang[#1], Bowei Zhou[#1], Xiao Yang[1], Jin Zhao[1], Jingjuan Hu[1], Yuqi Ding[1], Shuteng Zhan[1], Yifeng Yang[1], Jun Chen[1], Fu Zhang[1], Bingcheng Zhao[1], Fan Deng[1], Zebin Lin[1], Qishun Sun[1], Fangling Zhang[1], Zhiwen Yao[1], Weifeng Liu[1], Cai Li*[1] and Ke-Xuan Liu*[1]

*Corresponding author. Email:liukexuan705@163.com; licaisysu@163.com.

File list:

Supplementary Figures

Supplementary Fig. 1
Supplementary Fig. 2
Supplementary Fig. 3
Supplementary Fig. 4
Supplementary Fig. 5
Supplementary Fig. 6
Supplementary Fig. 7
Supplementary Fig. 8
Supplementary Fig. 9

Supplementary Tables

Supplementary Table 1
Supplementary Table 2
Supplementary Table 3
Supplementary Table 4

Supplementary Figures

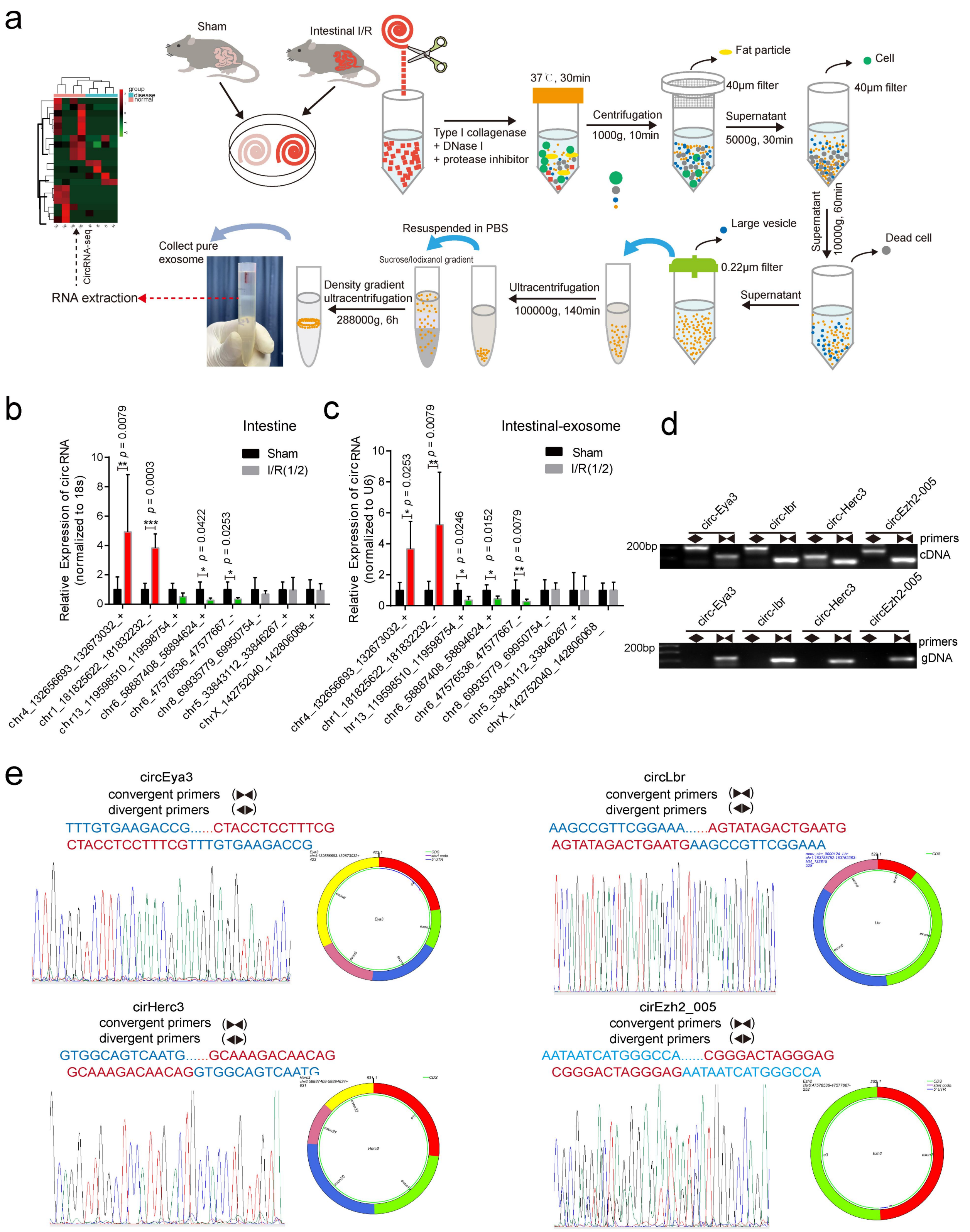

Supplementary Fig. 1. Identification of the top four differentially expressed circRNAs. (a) The experimental scheme of exosome extraction from intestinal tissue. **(b, c)** RT-qPCR assay to illustrate the accuracy of circRNA-seq data from exosomes and confirmation of the eight highly expressed circRNAs (n = 5 mice per group). **(d)** The amplification of circRNAs from cDNA or genomic DNA (gDNA) with divergent and convergent primers was shown by PCR. **(e)** Schematic illustration illustrating the generation of the top four putative target circRNAs from its host gene, then validated by Sanger sequencing. Data were presented as mean ± SD. The statistical tests are two-sided unless otherwise specified. For b and c, the data were analyzed by the two-tailed Student's t-test or Mann–Whitney test and presented as means ± SD. (* $p < 0.05$, * *$p < 0.01$, *** $p < 0.001$, ****$p < 0.0001$). Source data are provided as a Source data file.

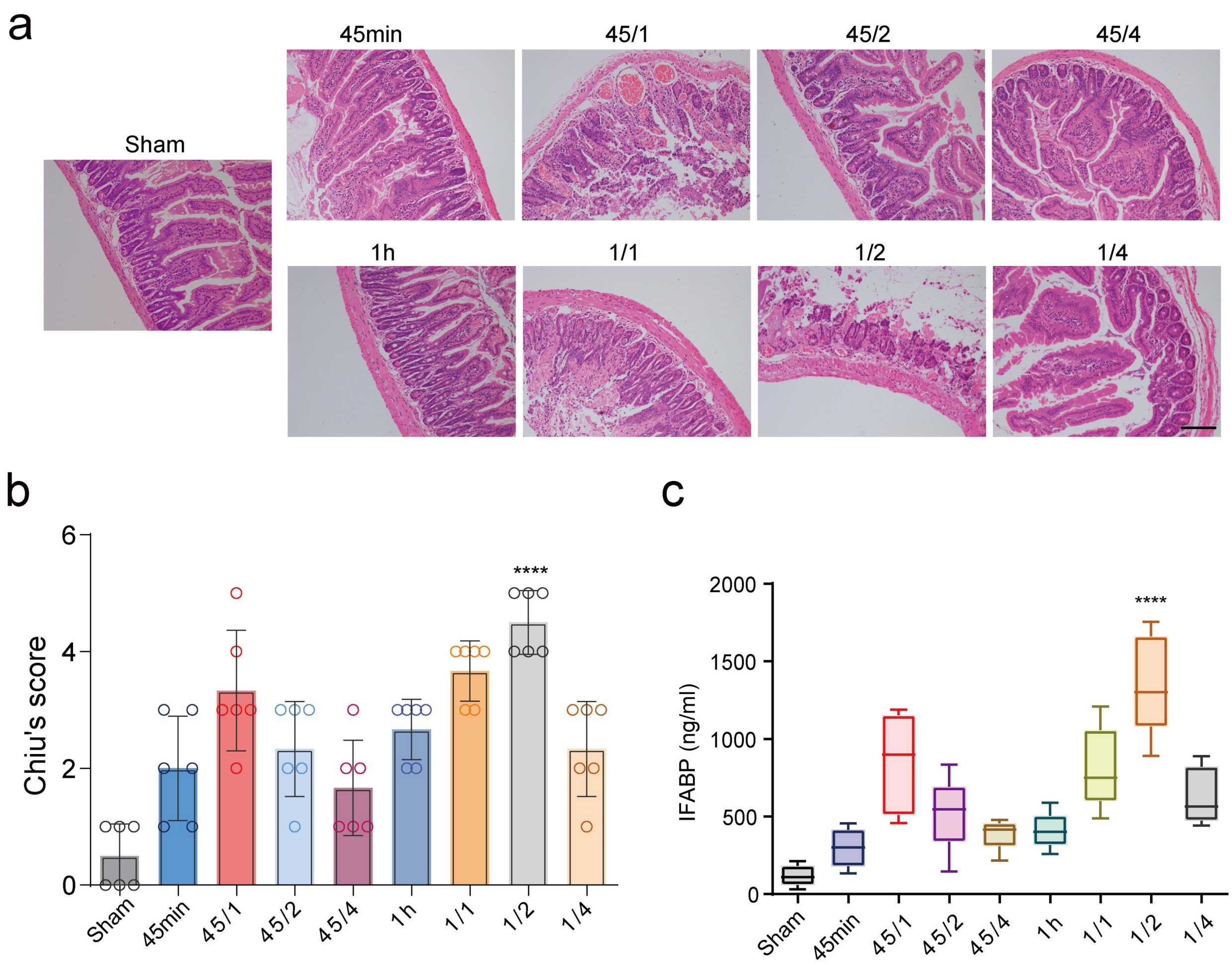

Supplementary Fig. 2. Intestinal I/R model mice were established at different time points. **(a)** Mice were subjected to intestinal I/R mode and underwent different time points of ischemia (45 min and 1h of ischemia) and different time points of reperfusion (1h, 2h, and 4h of reperfusion), the histopathological was estimated by the H&E staining (n = 6 mice per group). **(b)** Chui's score was used to assess the intestinal injury (n = 6 mice per group). Sham vs 1/2, $p < 0.0001$. (**c**) The ischemia-reperfusion-induced intestinal damage was evaluated by the serum I-FABP levels (n = 5 mice per group). Sham vs 1/2, $p < 0.0001$. Data were presented as mean ± SD. The statistical tests are two-sided unless otherwise specified. The data were analyzed by one-way ANOVA with Dunn's test. (* $p < 0.05$, ** $p < 0.01$, *** $p < 0.001$, ****$p < 0.0001$). Source data are provided as a Source data file.

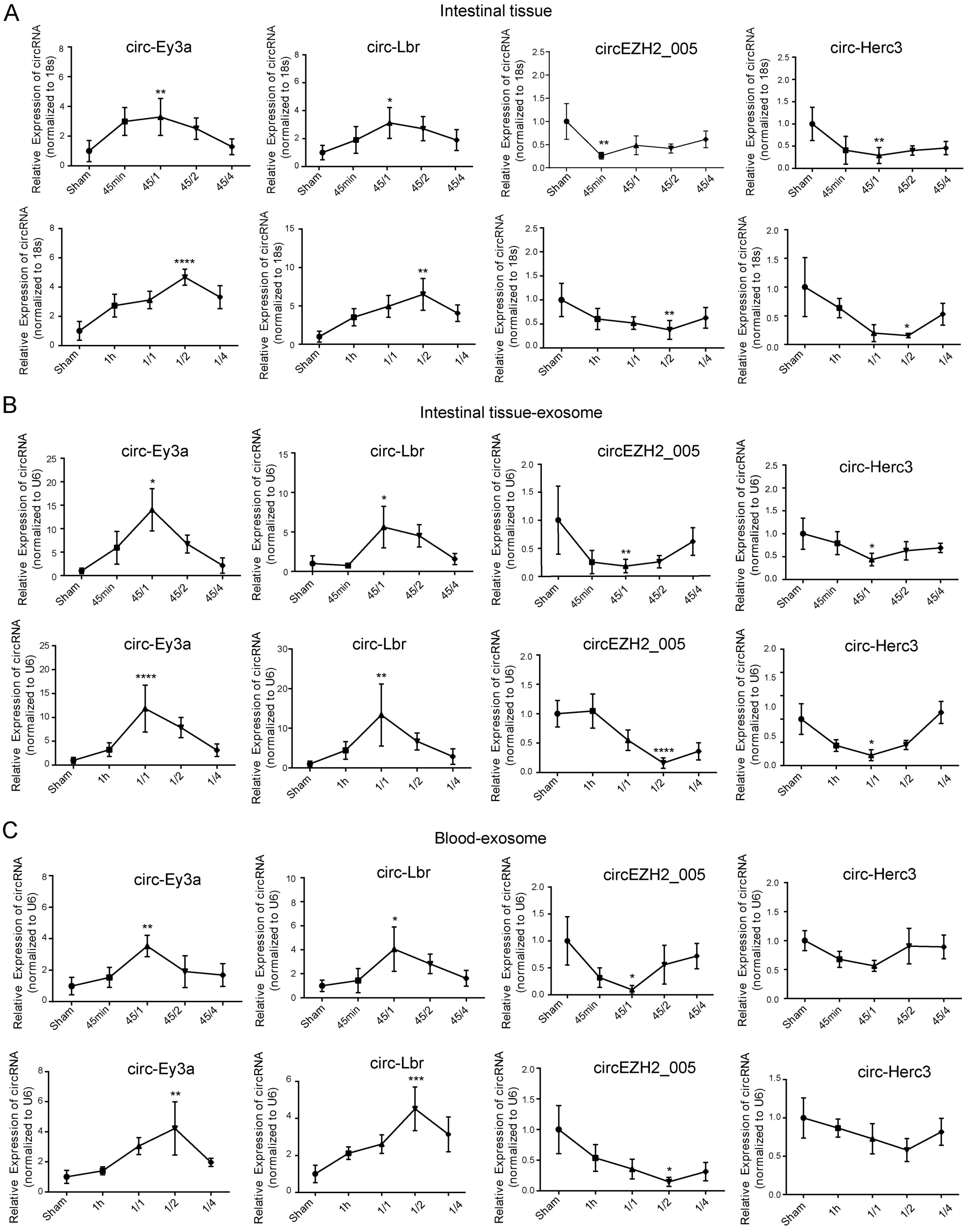
A
Intestinal tissue
circ-Ey3a
circ-Lbr
circEZH2_005
circ-Herc3
Relative Expression of circRNA (normalized to 18s)
Sham
45min
45/1
45/2
45/4
1h
1/1
1/2
1/4
B
Intestinal tissue-exosome
Relative Expression of circRNA (normalized to U6)
C
Blood-exosome

Supplementary Fig. 3. The expression of the top four differentially expressed circRNAs. (a) RT-qPCR for the expression of the top four differentially expressed circRNAs in intestinal tissues at different time points of intestinal I/R mice mode (n = 4 mice per group). circ-Ey3a, Sham vs 45/1, $p = 0.0067$. Sham vs 1/2, $p < 0.0001$; circ-Lbr, Sham vs 45/1, $p = 0.0199$. Sham vs 1/2, $p = 0.0050$; circEZH2_005, Sham vs 45/1, $p = 0.0021$. Sham vs 1/2, $p = 0.0062$; circ-Herc3, Sham vs 45/1, $p = 0.0036$. Sham vs 1/2, $p = 0.0239$. **(b)** Expression of the top four differentially expressed circRNAs in intestinal tissue exosomes at different time points of intestinal I/R mice mode (n = 4 mice per group). circ-Ey3a, Sham vs 45/1, $p = 0.0306$. Sham vs 1/2, $p < 0.0001$; circ-Lbr, Sham vs 45/1, $p = 0.0165$. Sham vs 1/1, $p = 0.0021$; circEZH2_005, Sham vs 45/1, $p = 0.0077$. Sham vs 1/2, $p < 0.0001$; circ-Herc3, Sham vs 45/1, $p = 0.0136$. Sham vs 1/2, $p = 0.0356$. (**c**) Expression of the top four differentially expressed circRNAs in plasma exosomes at different time points of intestinal I/R mice mode (n = 3 mice per group). circ-Ey3a, Sham vs 45/1, $p = 0.0059$. Sham vs 1/2, $p = 0.0035$; circ-Lbr, Sham vs 45/1, $p = 0.0190$. Sham vs 1/2, $p = 0.0007$; circEZH2_005, Sham vs 45/1, $p = 0.0114$. Sham vs 1/2, $p = 0.0104$. Data were presented as mean ± SD. The statistical tests are two-sided unless otherwise specified. The data were analyzed by one-way ANOVA with Dunnett's test or Kruskal-Wallis test with Dunn's test. (* $p < 0.05$, ** $p < 0.01$, *** $p < 0.001$, ****$p < 0.0001$). Source data are provided as a Source data file.

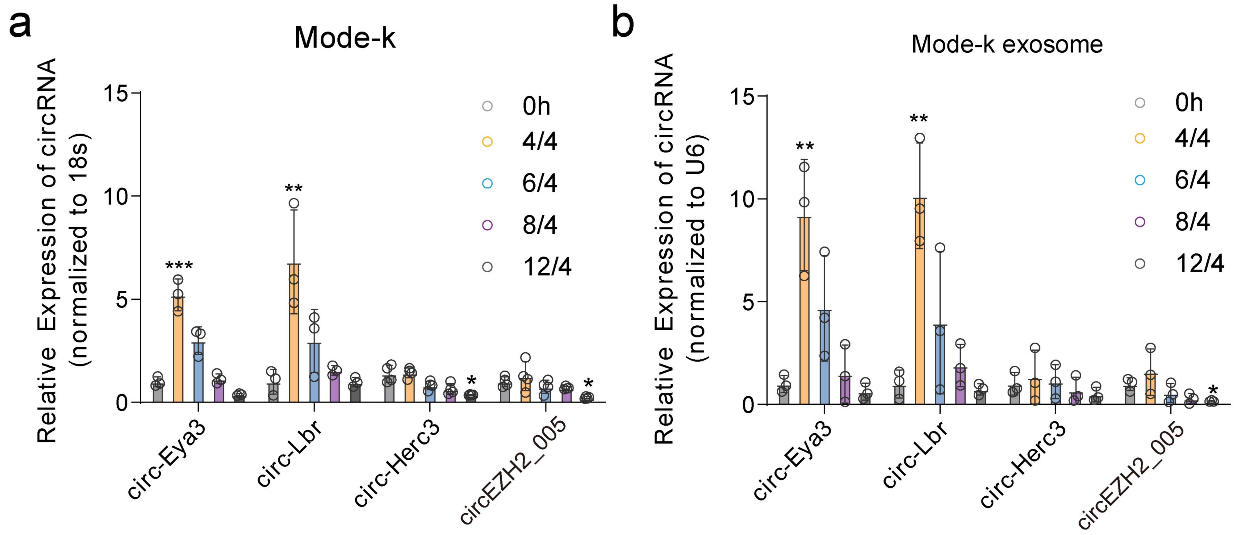

Supplementary Fig. 4. The expression of the top four differentially expressed circRNAs in cells. **(a)** RT-qPCR for the expression of the top four differentially expressed circRNAs in intestinal epithelial cell (Mode-K cell) and exosomes of cell supernatant subjected to different time points of hypoxia (4h, 6h, 8h, and 12h) and 4h of reoxygenation (n = 3-4 biological replicates). circ-Ey3a, 0h vs 4/4, $p = 0.008$; circ-Lbr, 0h vs 4/4, $p = 0.0177$; circ-Herc3, 0h vs 12/4, $p = 0.0161$; circEZH2_005, 0h vs 12/4, $p = 0.0305$. **(b)** RT-qPCR for the expression of the top four differentially expressed circRNAs in exosomes of cell supernatant subjected to different time points of hypoxia (4h, 6h, 8h, and 12h) and 4h of reoxygenation (n = 3 biological replicates). circ-Ey3a, 0h vs 4/4, $p = 0.0065$; circ-Lbr, 0h vs 4/4, $p = 0.0040$; circEZH2_005, 0h vs 12/4, $p = 0.0108$. Data were presented as mean ± SD. The statistical tests are two-sided unless otherwise specified. The data were analyzed by the two-tailed Student's t-test. (* $p < 0.05$, ** $p < 0.01$, *** $p < 0.001$, **** $p < 0.0001$). Source data are provided as a Source data file.

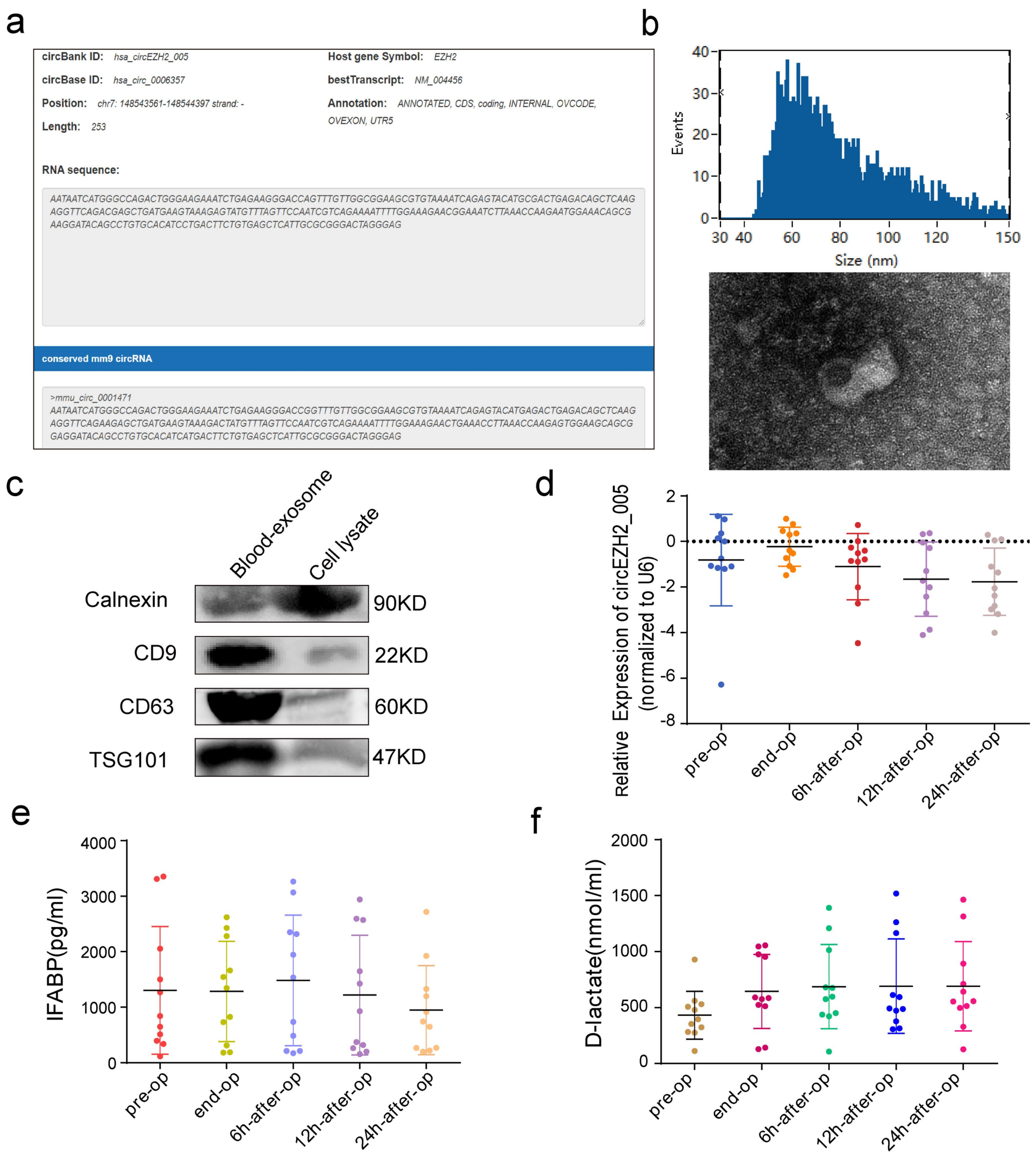
a
circBank ID: hsa_circEZH2_005
Host gene Symbol: EZH2
circBase ID: hsa_circ_0006357
bestTranscript: NM_004456
Position: chr7: 148543561-148544397 strand: -
Annotation: ANNOTATED, CDS, coding, INTERNAL, OVCODE, OVEXON, UTR5
Length: 253
RNA sequence:
AATAATCATGGGCCAGACTGGGAAGAAATCTGAGAAGGGACCAGTTTGTTGGCGGAAGCGTGTAAAATCAGAGTACATGCGACTGAGACAGCTCAAG
AGGTTCAGACGAGCTGATGAAGTAAAGAGTATGTTTAGTTCCAATCGTCAGAAAATTTTGGAAAGAACGGAAATCTTAAACCAAGAATGGAAACAGCG
AAGGATACAGCCTGTGCACATCCTGACTTCTGTGAGCTCATTGCGCGGGACTAGGGAG
conserved mm9 circRNA
>mmu_circ_0001471
AATAATCATGGGCCAGACTGGGAAGAAATCTGAGAAGGGACCGGTTTGTTGGCGGAAGCGTGTAAAATCAGAGTACATGAGACTGAGACAGCTCAAG
AGGTTCAGAAGAGCTGATGAAGTAAAGACTATGTTTAGTTCCAATCGTCAGAAAATTTTGGAAAGAACTGAAACCTTAAACCAAGAGTGGAAGCAGCG
GAGGATACAGCCTGTGCACATCATGACTTCTGTGAGCTCATTGCGCGGGACTAGGGAG
b
Events
40
30
20
10
0
30 40 60 80 100 120 150
Size (nm)
c
Blood-exosome
Cell lysate
Calnexin
90KD
CD9
22KD
CD63
60KD
TSG101
47KD
d
Relative Expression of circEZH2_005 (normalized to U6)
2
0
-2
-4
-6
-8
pre-op
end-op
6h-after-op
12h-after-op
24h-after-op
e
IFABP(pg/ml)
4000
3000
2000
1000
0
pre-op
end-op
6h-after-op
12h-after-op
24h-after-op
f
D-lactate(nmol/ml)
2000
1500
1000
500
0
pre-op
end-op
6h-after-op
12h-after-op
24h-after-op

Supplementary Fig. 5. Isolating and analyzing plasma exosomes from clinical patients. (**a**) Analysis of conservation of circEZH2_005 in humans and mice using circBank database. (**b**) The particle size and electron micrograph of plasma exosomes extracted by commercial kit. (**c**) The exosome-specific markers CD9, CD63, TSG101, and Calnexin were shown by western blot. (**d**) RT-qPCR analysis of exosomal circEZH2_005 expression in the plasma of patients without intestinal I/R injury in preoperative, end operative, 6 hours after operative, 12 hours after operative, and 24 hours after operative (n = 11 in controls). (**e**, **f**) Plasma I-FABP and D-lactate levels were assessed by ELISA (n = 11 in controls). Data were presented as mean ± SD. The statistical tests are two-sided unless otherwise specified. For d-f, the data were analyzed by Friedman test. (* $p < 0.05$, ** $p < 0.01$, *** $p < 0.001$). Source data are provided as a Source data file.

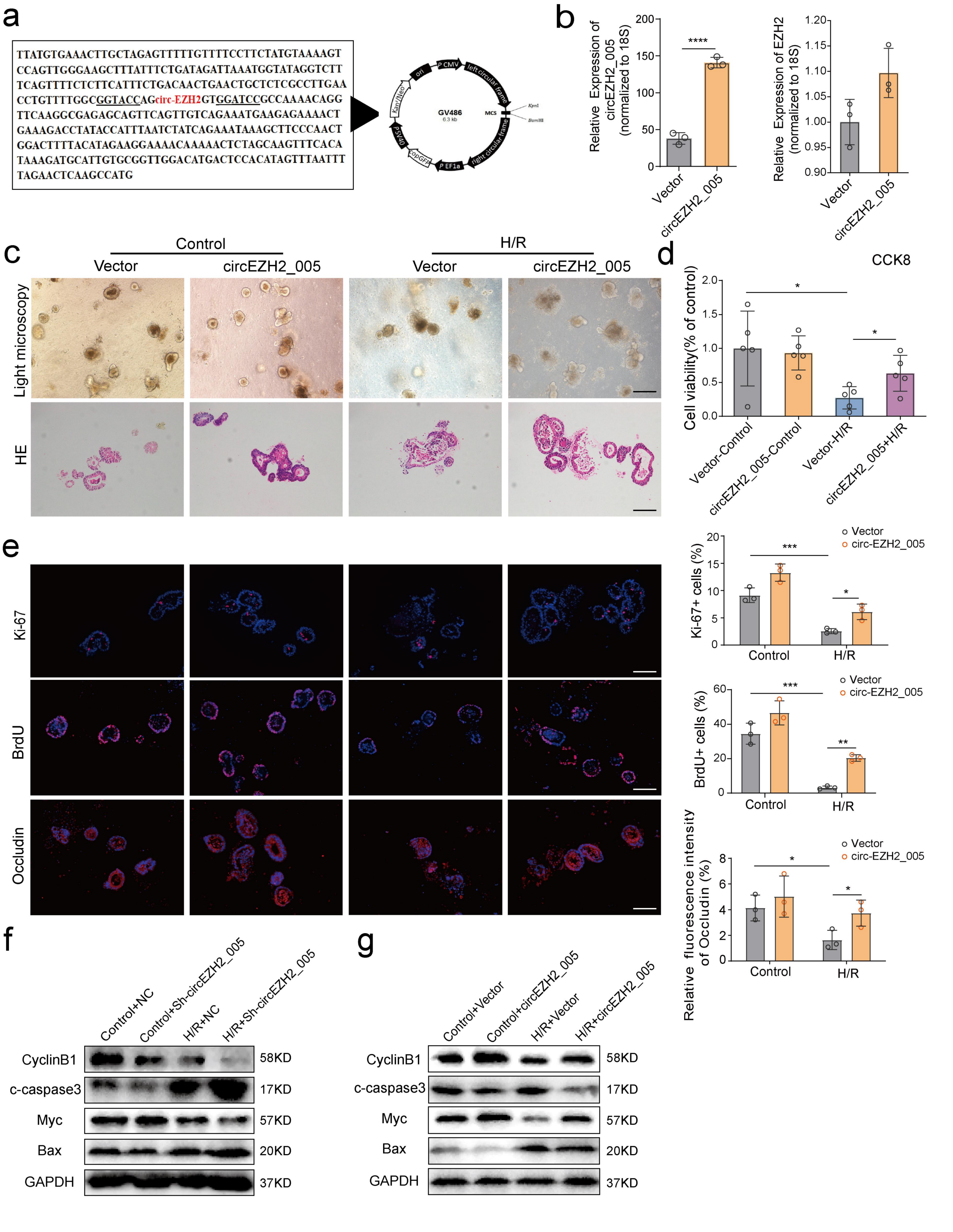
a
TTATGTGAAACTTGCTAGAGTTTTTGTTTTCCTTCTATGTAAAAGT
CCAGTTGGGAAGCTTTATTTCTGATAGATTAAATGGTATAGGTCTT
TCAGTTTTCTCTTCATTTCTGACAACTGAACTGCTCTCGCCTTGAA
CCTGTTTTGGCGGTACCAGcirc-EZH2GTGGATCCGCCAAAACAGG
TTCAAGGCGAGAGCAGTTCAGTTGTCAGAAATGAAGAGAAAACT
GAAAGACCTATACCATTTAATCTATCAGAAATAAAGCTTCCCAACT
GGACTTTTACATAGAAGGAAAACAAAAACTCTAGCAAGTTTCACA
TAAAGATGCATTGTGCGGTTGGACATGACTCCACATAGTTTAATTT
TAGAACTCAAGCCATG
GV486
MCS
b
Relative Expression of circEZH2_005 (normalized to 18S)
****
Vector
circEZH2_005
Relative Expression of EZH2 (normalized to 18S)
c
Control
H/R
Vector
circEZH2_005
Light microscopy
HE
d
CCK8
Cell viability(% of control)
Vector-Control
circEZH2_005-Control
Vector-H/R
circEZH2_005+H/R
e
Ki-67
BrdU
Occludin
Ki-67+ cells (%)
BrdU+ cells (%)
Relative fluorescence intensity of Occludin (%)
Vector
circ-EZH2_005
Control
H/R
f
Control+NC
Control+Sh-circEZH2_005
H/R+NC
H/R+Sh-circEZH2_005
CyclinB1
c-caspase3
Myc
Bax
GAPDH
58KD
17KD
57KD
20KD
37KD
g
Control+Vector
Control+circEZH2_005
H/R+Vector
H/R+circEZH2_005
CyclinB1
c-caspase3
Myc
Bax
GAPDH
58KD
17KD
57KD
20KD
37KD

Supplementary Fig. 6. **circEZH2_005 protects intestinal organoids from H/R injury in vitro**. **(a)** The overexpression sequence of circEZH2_005 (the two end sequences of circEZH2_005 are partial concyclic sequences) was connected to the gv486 vector to construct circEZH2_005 overexpression plasmid, and then packaged on adenovirus vector. **(b)** RT-qPCR analysis of circEZH2_005 overexpression and EZH2 expression in circEZH2_005 -adenovirus transfected cells (n = 3 biological replicates). Vector vs circEZH2_005, $p < 0.0001$. **(c)** Organoid morphology was observed by light microscopy and HE staining, scale bar is 100μm (n = 3 biological replicates). **(d)** The organoid viability was analyzed by CCK-8 (n = 5 biological replicates). Control Vector vs H/R Vector, $p = 0.0393$. H/R Vector vs H/R circEZH2_005, $p = 0.0320$. **(e)** Immunofluorescence staining for the Ki-67, BrdU, and occludin in the organoids for proliferation analysis, scale bar is 100μm (n = 3 biological replicates). Ki-67, Control Vector vs H/R Vector, $p = 0.0006$. H/R Vector vs H/R circEZH2_005, $p = 0.0249$; BrdU, Control Vector vs H/R Vector, $p = 0.0001$. H/R Vector vs H/R circEZH2_005, $p = 0.0058$; Occludin, Control Vector vs H/R Vector, $p = 0.0262$. H/R Vector vs H/R circEZH2_005, $p = 0.0458$. **(f)** Western blot was conducted to analyze apoptotic and proliferation-related proteins induced by H/R and circEZH2_005 interference (n = 3 biological replicates). **(g)** Western blot was conducted to analyze apoptotic and proliferation-related proteins induced by H/R and circEZH2_005 overexpression (n = 3 biological replicates). Data were presented as mean ± SD. The statistical tests are two-sided unless otherwise specified. For b and d, the data were analyzed by the unpaired two-tailed Student's t-test. For e, one-way ANOVA with Dunnett's test. (* $p < 0.05$, ** $p < 0.01$, *** $p < 0.001$, ****$p < 0.0001$). Source data are provided as a Source data file.

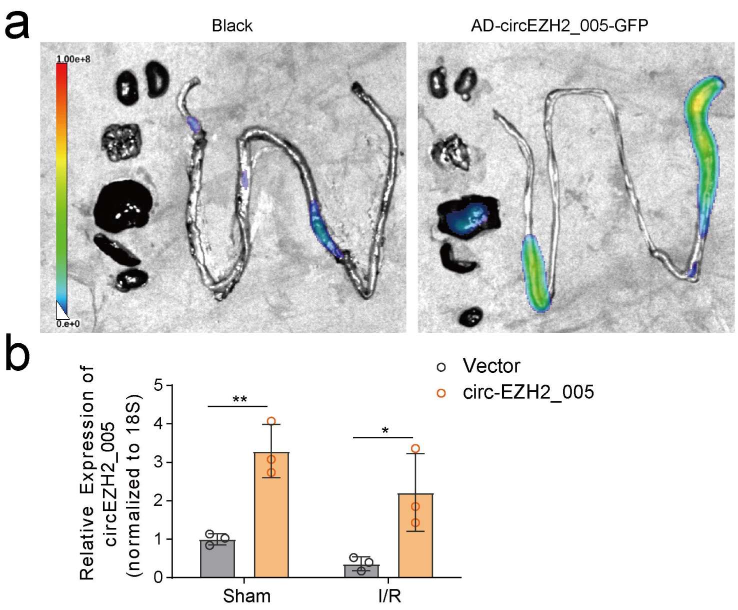

Supplementary Fig. 7. Effect of circEZH2_005 overexpression in the mouse. (**a**) Fluorescence in vivo imaging was used to detect the overexpression position of circEZH2_005 adenovirus vector in mice after microinjecting with circEZH2_005 adenovirus. (**b**) RT-qPCR analysis of circEZH2_005 overexpression in intestinal tissues and intestinal crypt cell (n = 3 mice per group). Sham Vector vs Sham circEZH2_005, $p = 0.0049$. I/R Vector vs I/R circEZH2_005, $p = 0.0358$. Data were presented as mean ± SD. The statistical tests are two-sided unless otherwise specified. The data were analyzed by the Student's t-test. (* $p < 0.05$, ** $p < 0.01$, *** $p < 0.001$, **** $p < 0.0001$). Source data are provided as a Source data file.

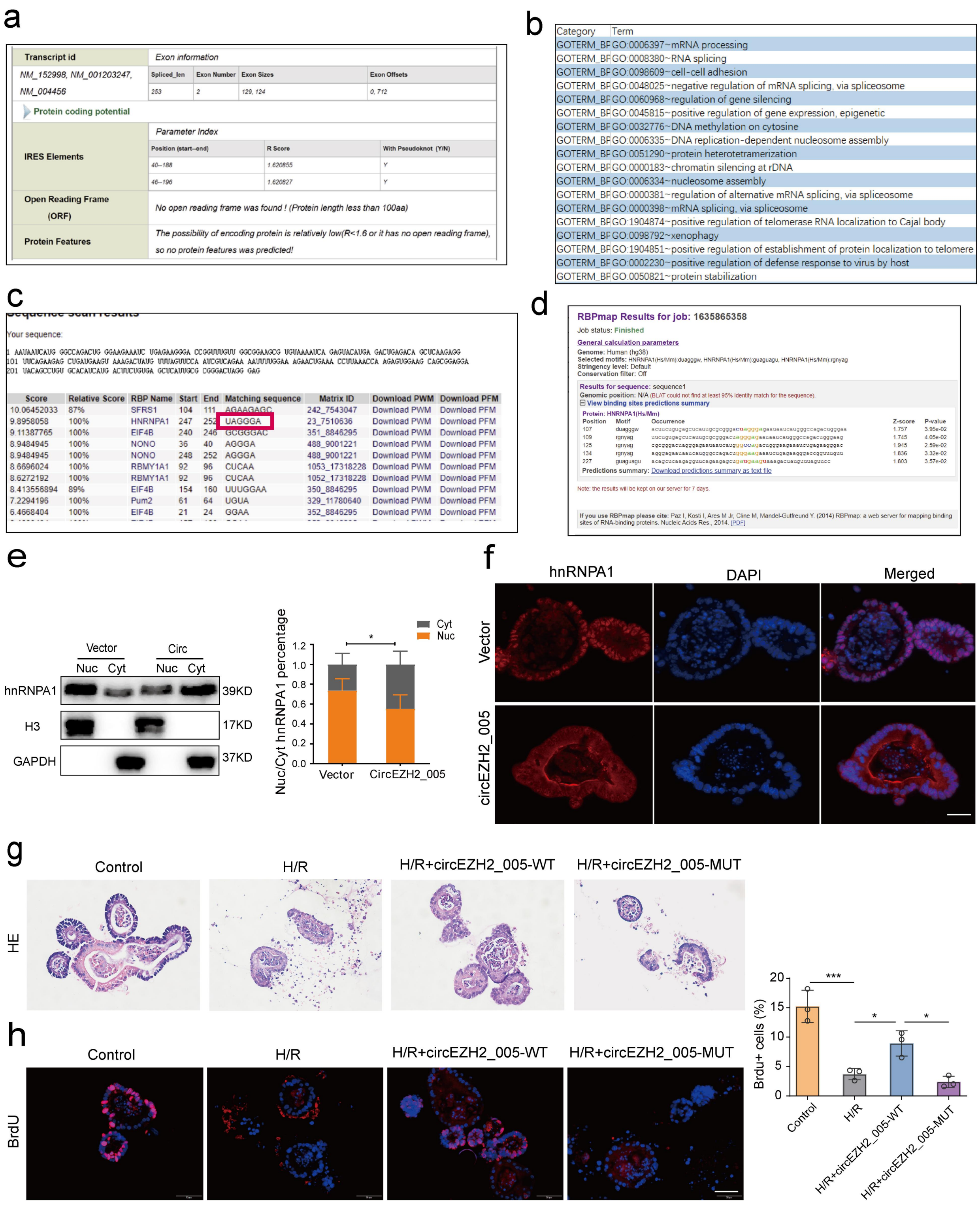
a
Transcript id
Exon information
NM_152998, NM_001203247, NM_004456
Spliced_len
Exon Number
Exon Sizes
Exon Offsets
253
2
129, 124
0, 712
Protein coding potential
Parameter Index
IRES Elements
Position (start--end)
R Score
With Pseudoknot (Y/N)
40--188
1.620855
Y
46--196
1.620827
Y
Open Reading Frame (ORF)
No open reading frame was found ! (Protein length less than 100aa)
Protein Features
The possibility of encoding protein is relatively low(R<1.6 or it has no open reading frame), so no protein features was predicted!
b
Category
Term
GOTERM_BP GO:0006397~mRNA processing
GOTERM_BP GO:0008380~RNA splicing
GOTERM_BP GO:0098609~cell-cell adhesion
GOTERM_BP GO:0048025~negative regulation of mRNA splicing, via spliceosome
GOTERM_BP GO:0060968~regulation of gene silencing
GOTERM_BP GO:0045815~positive regulation of gene expression, epigenetic
GOTERM_BP GO:0032776~DNA methylation on cytosine
GOTERM_BP GO:0006335~DNA replication-dependent nucleosome assembly
GOTERM_BP GO:0051290~protein heterotetramerization
GOTERM_BP GO:0000183~chromatin silencing at rDNA
GOTERM_BP GO:0006334~nucleosome assembly
GOTERM_BP GO:0000381~regulation of alternative mRNA splicing, via spliceosome
GOTERM_BP GO:0000398~mRNA splicing, via spliceosome
GOTERM_BP GO:1904874~positive regulation of telomerase RNA localization to Cajal body
GOTERM_BP GO:0098792~xenophagy
GOTERM_BP GO:1904851~positive regulation of establishment of protein localization to telomere
GOTERM_BP GO:0002230~positive regulation of defense response to virus by host
GOTERM_BP GO:0050821~protein stabilization
c
Your sequence:
Score
Relative Score
RBP Name
Start
End
Matching sequence
Matrix ID
Download PWM
Download PFM
10.06452033 87% SFRS1 104 111 AGAAGAGC 242_7543047 Download PWM Download PFM
9.8958058 100% HNRNPA1 247 252 UAGGGA 23_7510636 Download PWM Download PFM
9.11387765 100% EIF4B 240 246 GCGGGAC 351_8846295 Download PWM Download PFM
8.9484945 100% NONO 36 40 AGGGA 488_9001221 Download PWM Download PFM
8.9484945 100% NONO 248 252 AGGGA 488_9001221 Download PWM Download PFM
8.6696024 100% RBMY1A1 92 96 CUCAA 1053_17318228 Download PWM Download PFM
8.6272192 100% RBMY1A1 92 96 CUCAA 1052_17318228 Download PWM Download PFM
8.413556894 89% EIF4B 154 160 UUUGGAA 350_8846295 Download PWM Download PFM
7.2294196 100% Pum2 61 64 UGUA 329_11780640 Download PWM Download PFM
6.4668404 100% EIF4B 21 24 GGAA 352_8846295 Download PWM Download PFM
d
RBPmap Results for job: 1635865358
Job status: Finished
General calculation parameters
Genome: Human (hg38)
Stringency level: Default
Conservation filter: Off
Results for sequence: sequence1
View binding sites predictions summary
Protein: HNRNPA1(Hs/Mm)
Position
Motif
Occurrence
Z-score
P-value
Predictions summary: Download predictions summary as text file
Note: the results will be kept on our server for 7 days.
e
Vector
Circ
Nuc
Cyt
hnRNPA1
H3
GAPDH
39KD
17KD
37KD
Nuc/Cyt hnRNPA1 percentage
Cyt
Nuc
*
1.2
1.0
0.8
0.6
0.4
0.2
0.0
Vector
CircEZH2_005
f
hnRNPA1
DAPI
Merged
Vector
circEZH2_005
g
Control
H/R
H/R+circEZH2_005-WT
H/R+circEZH2_005-MUT
HE
h
Control
H/R
H/R+circEZH2_005-WT
H/R+circEZH2_005-MUT
BrdU
Brdu+ cells (%)
20
15
10
5
0
***
*
*
Control
H/R
H/R+circEZH2_005-WT
H/R+circEZH2_005-MUT

Supplementary Fig. 8. Bioinformatics database analysis of circEZH2_005-bound proteins. **(a)** The CircRNADb website analyzed whether circEZH2_005 was able to translate the protein. **(b)** GO enrichment analysis was used to cluster and characterize the biological processes of circ-EZH2-binding proteins. **(c)** Proteins for circEZH2_005 binding are predicted by the RBPDB database. **(d)** Association between circEZH2_005 and hnRNPA1 was predicted by the RBPmap database. **(e)** Western blot was conducted to analyze the expression of cytoplasmic (Cyt) and nuclear (Nuc) hnRNPA1 in circEZH2_005-overexpressed intestinal crypt stem cells (n = 5 biological replicates). Cyt, Vector vs circEZH2_005, $p = 0.0474$. **(f)** Immunofluorescent of hnRNPA1 (red) in the organoids after overexpressing circEZH2_005. DAPI (blue) for nuclear staining, scale bar = 50μm. **(g)** HE staining was used to analyze whether a mutated version of circEZH2_005-overexpressed virus could alleviate organoid damage induced by H/R, Scale bars is 100μm (n = 3 biological replicates). **(h)** Immunofluorescence staining was used to analyze whether a mutated version of circEZH2_005-overexpressed virus could alleviate organoid proliferation induced by H/R , Scale bars is 100μm (n = 3 biological replicates). Control vs H/R, $p = 0.0003$. H/R vs H/R + circEZH2_005-WT, $p = 0.0375$. H/R + circEZH2_005-WT vs H/R + circEZH2_005-MUT, $p = 0.0116$. Data were presented as mean ± SD. The statistical tests are two-sided unless otherwise specified. For e, the data were analyzed by the unpaired two-tailed Student's t-test. For h, one-way ANOVA with Tukey's test. (* $p < 0.05$, ** $p < 0.01$, *** $p < 0.001$, ****$p < 0.0001$). Source data are provided as a Source data file.

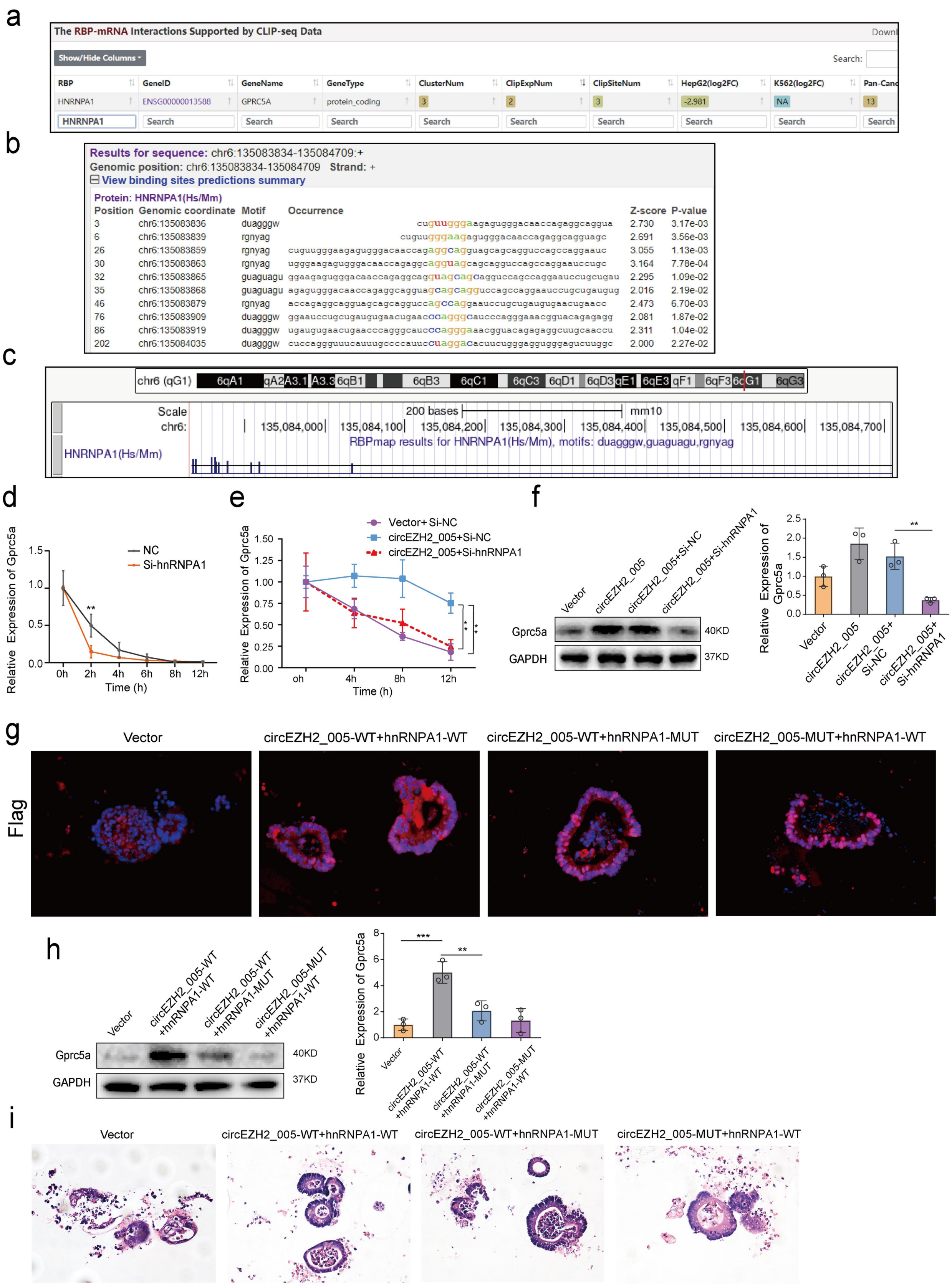

a
The RBP-mRNA Interactions Supported by CLIP-seq Data
Show/Hide Columns
Search:
RBP
GeneID
GeneName
GeneType
ClusterNum
ClipExpNum
ClipSiteNum
HepG2(log2FC)
K562(log2FC)
HNRNPA1
ENSG00000013588
GPRC5A
protein_coding
3
2
3
-2.981
NA
13
b
Results for sequence: chr6:135083834-135084709:+
Genomic position: chr6:135083834-135084709 Strand: +
View binding sites predictions summary
Protein: HNRNPA1(Hs/Mm)
Position Genomic coordinate Motif Occurrence Z-score P-value
3 chr6:135083836 duagggw 2.730 3.17e-03
6 chr6:135083839 rgnyag 2.691 3.56e-03
26 chr6:135083859 rgnyag 3.055 1.13e-03
30 chr6:135083863 rgnyag 3.164 7.78e-04
32 chr6:135083865 guaguagu 2.295 1.09e-02
35 chr6:135083868 guaguagu 2.016 2.19e-02
46 chr6:135083879 rgnyag 2.473 6.70e-03
76 chr6:135083909 duagggw 2.081 1.87e-02
86 chr6:135083919 duagggw 2.311 1.04e-02
202 chr6:135084035 duagggw 2.000 2.27e-02
c
chr6 (qG1)
Scale 200 bases mm10
chr6: 135,084,000 135,084,100 135,084,200 135,084,300 135,084,400 135,084,500 135,084,600 135,084,700
RBPmap results for HNRNPA1(Hs/Mm), motifs: duagggw,guaguagu,rgnyag
HNRNPA1(Hs/Mm)
d
NC
Si-hnRNPA1
Relative Expression of Gprc5a
Time (h)
0h 2h 4h 6h 8h 12h
e
Vector+ Si-NC
circEZH2_005+Si-NC
circEZH2_005+Si-hnRNPA1
Relative Expression of Gprc5a
Time (h)
oh 4h 8h 12h
f
Vector
circEZH2_005
circEZH2_005+Si-NC
circEZH2_005+Si-hnRNPA1
Gprc5a 40KD
GAPDH 37KD
Relative Expression of Gprc5a
g
Vector
circEZH2_005-WT+hnRNPA1-WT
circEZH2_005-WT+hnRNPA1-MUT
circEZH2_005-MUT+hnRNPA1-WT
Flag
h
Gprc5a 40KD
GAPDH 37KD
Relative Expression of Gprc5a
i
Vector
circEZH2_005-WT+hnRNPA1-WT
circEZH2_005-WT+hnRNPA1-MUT
circEZH2_005-MUT+hnRNPA1-WT

Supplementary Fig. 9. circEZH2_005 prevents intestinal I/R injury by stabilizing G*prc5a*. (a) The starBase data predicted that hnRNPA1 can bind to the mRNA of Gprc5a. **(b)** The hnRNPA1 binding sites in the Gprc5a 3'UTR region predicted by RBPmap. **(c)** The bottom blue vertical lines represented the hnRNPA1 binding sites in 3'UTR of Gprc5a mRNA. **(d)** The rate of degradation of the G*prc5a* was assessed after transfection with hnRNPA1 siRNA in intestinal crypt stem cells (n = 5 biological replicates). 2h, NC vs Si-hnRNPA1, $p = 0.0021$. **(e)** The degradation rate of Gprc5a mRNA in intestinal crypt stem cells was evaluated after circEZH2_005 overexpression and hnRNPA1 interference simultaneously (n = 3 biological replicates). Vector + Si-NC vs circEZH2_005 + Si-NC, $p = 0.001$. circEZH2_005 + Si-hnRNPA1 vs circEZH2_005 + Si-NC, $p = 0.0019$. **(f)** Western blot assay examined whether interfering with hnRNPA1 could reverse the effect of circEZH2_005 on Gprc5a protein expression (n = 3 biological replicates). circ-EZH2_005 + Si-NC vs H/R + circ-EZH2_005 + Si-hnRNPA1, $p = 0.0069$. **(g)** Flag antibody was used to detect the localization of HnRNPA1 after co-transfection of a mutated version of circEZH2_005 and a mutation of the hnRNPA1, Scale bars is 100μm. **(h)** Western blot assay was used to detect Gprc5a protein expression (n = 3 biological replicates). Vector vs circEZH2_005-WT + hnRNPA1 WT, $p = 0.0009$. circEZH2_005-WT + hnRNPA1 WT vs circEZH2_005-WT + hnRNPA1 MUT, $p = 0.0064$. **(i)** HE staining was used to analyze the organoid damage induced by H/R, Scale bars is 100μm (n = 3 biological replicates). Data were presented as mean ± SD. The statistical tests are two-sided unless otherwise specified. For d, the data were analyzed by the two-tailed unpaired t test. For e, f and h, one-way ANOVA with Tukey's test. (* $p < 0.05$, ** $p < 0.01$, *** $p < 0.001$, ****$p < 0.0001$). Source data are provided as a Source data file.

Supplementary Tables

Supplementary Table 1. Baseline characteristics for AGI on the first operative day

	Total (n=50)	AGI < 2 (n=11)	AGI ≥2 (n=39)	p-Value
Age, median (IQR)	58.0 (53.0-65.5)	53.0 (39.0-56.0)	59.0 (55.0-70.0)	0.011
Sex, n (%)				0.594
Male	17 (34)	3 (27)	14 (36)	
Female	33 (66)	8 (73)	25 (64)	
BMI (kg/m2), mean ± SD	23.8±0.5	22.2±1.2	24.2±0.6	0.095
ASA, n (%)				0.737
III	44 (88)	10 (91)	34 (87)	
IV	6 (12)	1 (9)	5 (13)	
Duration of anesthesia (min), median (IQR)	329.0 (309.3-365.0)	318.0 (306.0-355.0)	330.0 (310.0-365.0)	0.482
Duration of surgery (min), median (IQR)	272.5 (244.5-302.0)	273.0 (242.0-291.0)	272.0 (245.0-302.0)	0.815
CPB total time (min), median (IQR)	135.0 (94.8-164.8)	106.0 (93.0-156.0)	142.0 (95.0-167.0)	0.426
**Postoperative**				
Duration of postoperative intubation (min), median (IQR)	1227.5 (1039.3-1665.0)	1077.0 (470.0-1270.0)	1245.0 (1053.0-2650.0)	0.015
Duration of postoperative fasting (min), median (IQR)	1622.5 (1393.8-2043.3)	1372.0 (1260.0-1580.0)	1732.0 (1478.0-2540.0)	0.006

Abbreviations: IQR, interquartile range; SD, standard deviation; AGI, acute gastrointestinal injury; BMI, body mass index; ASA: American Society of Anesthesiologists physical status; CPB: cardiopulmonary bypass.

Supplementary Table 2. The sequences of primers used for qRT-PCR

Gene name	Sequence (5'-3')
CircEZH2_005 (forward)	TTTAGTTCCAATCGTCAG
CircEZH2_005 (reverse)	CTCAGATTTCTTCCCAGT
Circ-Eya3 (forward)	ACCCAAACTTACGGACTA
Circ-Eya3 (reverse)	CTCAGGCTTCTCATCACT
Circ-Lbr2 (forward)	CTGCTCCACTTCCCTCCA
Circ-Lbr2 (reverse)	CACGCTGACGCTGTTTCC
Circ-Herc3 (forward)	ACAACAGGTGGCAGTCAA
Circ-Herc3 (reverse)	ACAAGGTGGTTCCTACGG
U6 (forward)	AACGCTTCACGAATTTGCGT
U6(reverse)	GCTTCGGCAGCACATATACTAA
Gprc5a (forward)	TACAACTGCCCCTAGCGGT
Gprc5a (reverse)	TGAGGAAAACGAGTGCAAACAT
hnRNPA1(forward)	GAAACAACCGACGAGAGTCTG
hnRNPA1 (reverse)	TGTGTGGTCTTGCATTCATGG
TNF-α (forward)	CCCTCACACTCAGATCATCTTCT
TNF-α (reverse)	GCTACGACGTGGGCTACAG
IL-6 (forward)	TAGTCCTTCCTACCCCAATTTCC
IL-6 (reverse)	TTGGTCCTTAGCCACTCCTTC
EZH2 (forward)	AGTGACTTGGATTTTCCAGCAC
EZH2 (reverse)	AATTCTGTTGTAAGGGCGACC
Hspa12a (forward)	TCGGGGACACAGGAATAACAC
Hspa12a (reverse)	GGTAAAGCTGTAGGCATAGCC
Cldn4 (forward)	GTCCTGGGAATCTCCTTGGC
Cldn4 (reverse)	TCTGTGCCGTGACGATGTTG
Eno3 (forward)	CACAGCCAAGGGTCGATTCC
Eno3 (reverse)	CCCAGGTATCGTGCTTTGTCT
Tmem146(forward)	ACACACACAAGCATCTACTTTGA
Tmem146 (reverse)	AGAGGACTGCACATTCACTGT

Jun (forward)	TGTGCCCCAAGAACGTGAC
Jun (reverse)	CCGGGTTGAAGTTGCTGAG
Gp1bb (forward)	TGACCGGCAACAACCTGAC
Gp1bb (reverse)	CAGCAGAGTAGACCGGGTG
Elmo1 (forward)	TGTAACCCACGATTTGCAGGA
Elmo1 (reverse)	TGCAGACATCCGTGAGTGTTC
Tlco2 (forward)	GCCTCTGGGATGGATGAGTCT
Tlco2 (reverse)	GCATGAGGATAAACGGGTAGGG
Lama3 (forward)	CTGTGACTACTGCAATTCTGAGG
Lama3 (reverse)	CAAGGTGAGGTTGACTTGATTGT
18S (forward)	ACACGGACAGGATTGACAGA
18S (reverse)	GGACATCTAAGGGCATCACA

Supplementary Table 3. Sequences of circEZH2_005 used for RNA pulldown analysis in the study

Gene name	Sequences (5'-3')
mmu_circ_0001471(3bio)_ChIRP Probe_1	GATTATTCTCCCTAGTCCCG
mmu_circ_0001471(3bio)_ChIRP Probe_2	ATGATTATTCTCCCTAGTCC
mmu_circ_0001471(3bio)_ChIRP Probe_3	CCATGATTATTCTCCCTAGT
mmu_circ_0001471(3bio)_Scramble ChIRP NC_1	CCTCGCAGTTGTTTACACCT

Supplementary Table 4. Si-RNAs were used in the study.

Gene name	Sequence
si-mmu_circ_0001471_001	CGGGACTAGGGAGAATAAT
si-mmu_circ_0001471_002	GGACTAGGGAGAATAATCA
si-mmu_circ_0001471_003	CTAGGGAGAATAATCATGG
si-hnRNPA1-1	GGAUGGAAGAGUUGUGGAATT
si-hnRNPA1-2	GAGGGCUGAGCUUCGAAACAATT
si-hnRNPA1-3	GAACAUCACCUACGAGAUUAUTT
si-Gprc5a-1	UCACCUUCGCCUUCAUCAUCATT
si-Gprc5a-2	CUGGAUCGUUCUGCUCCUGAUTT
si-Gprc5a-3	CAGGACCAACGUCAAUGUCUUTT
