# Supplementary material for: Exosomal circEZH2_005, an intestinal injury biomarker, alleviates intestinal ischemia/reperfusion injury by mediating Gprc5a signaling
Source: Nat Commun. 2023 Sep 6;14:5437. doi: 10.1038/s41467-023-41147-3 (PMC10482849; doi:10.1038/s41467-023-41147-3)
Supplement: Supplementary file 3 — Reporting Summary [file 41467_2023_41147_MOESM3_ESM.pdf]

nature portfolio

Corresponding author(s): Cai Li , Ke-Xuan Liu

Last updated by author(s): Aug 15, 2023

Reporting Summary

Nature Portfolio wishes to improve the reproducibility of the work that we publish. This form provides structure for consistency and transparency in reporting. For further information on Nature Portfolio policies, see our Editorial Policies and the Editorial Policy Checklist.

Statistics

For all statistical analyses, confirm that the following items are present in the figure legend, table legend, main text, or Methods section.

n/a	Confirmed	
☐	☒	The exact sample size (*n*) for each experimental group/condition, given as a discrete number and unit of measurement
☐	☒	A statement on whether measurements were taken from distinct samples or whether the same sample was measured repeatedly
☐	☒	The statistical test(s) used AND whether they are one- or two-sided<br>*Only common tests should be described solely by name; describe more complex techniques in the Methods section.*
☒	☐	A description of all covariates tested
☐	☒	A description of any assumptions or corrections, such as tests of normality and adjustment for multiple comparisons
☐	☒	A full description of the statistical parameters including central tendency (e.g. means) or other basic estimates (e.g. regression coefficient) AND variation (e.g. standard deviation) or associated estimates of uncertainty (e.g. confidence intervals)
☐	☒	For null hypothesis testing, the test statistic (e.g. *F*, *t*, *r*) with confidence intervals, effect sizes, degrees of freedom and *P* value noted<br>*Give P values as exact values whenever suitable.*
☒	☐	For Bayesian analysis, information on the choice of priors and Markov chain Monte Carlo settings
☒	☐	For hierarchical and complex designs, identification of the appropriate level for tests and full reporting of outcomes
☐	☒	Estimates of effect sizes (e.g. Cohen's *d*, Pearson's *r*), indicating how they were calculated

Our web collection on statistics for biologists contains articles on many of the points above.

Software and code

Policy information about availability of computer code

Data collection	Data were collected using various instruments and equipments such as fluorescence imaging system(Olympus), ABI Q6 Real-Time PCR System(Thermo Fisher Scientific), Tanon 3500 system, FlowJo (Tree Star Inc.), The NovaSeq 6000 System with PE150 mode.
Data analysis	GraphPad Prism 8.01 software(GraphPadSoftware,Inc.,LaJolla,CA,USA), ImageJ (version 1.52), FlowJo (Tree Star Inc.).

For manuscripts utilizing custom algorithms or software that are central to the research but not yet described in published literature, software must be made available to editors and reviewers. We strongly encourage code deposition in a community repository (e.g. GitHub). See the Nature Portfolio guidelines for submitting code & software for further information.

Data

Policy information about availability of data

All manuscripts must include a data availability statement. This statement should provide the following information, where applicable:

- Accession codes, unique identifiers, or web links for publicly available datasets
- A description of any restrictions on data availability
- For clinical datasets or third party data, please ensure that the statement adheres to our policy

Data and materials availability:
All data needed to evaluate the conclusions of the paper are presented in the paper and/or the Supplementary Materials. RNA-sequencing data that supported the

findings of this study have been deposited in GEO (https://www.ncbi.nlm.nih.gov/geo/info/linking.html.) GSE227355, and GSE227518. The mass spectrometry proteomics data have been deposited to the ProteomeXchange Consortium (http://proteomecentral.proteomexchange.org) via the iProX partner repository with the dataset identifier PXD040925. Source data are
provided with this paper.

Research involving human participants, their data, or biological material

Policy information about studies with human participants or human data. See also policy information about sex, gender (identity/presentation), and sexual orientation and race, ethnicity and racism.

Reporting on sex and gender	Only information on sex was collected for this study. Information is provided in Supplementary Table 1. Baseline characteristics for AGI on the first operative day , a summary is given in the 'Population characteristics' field below. Gender was not collected for this study.
Reporting on race, ethnicity, or other socially relevant groupings	No information about the race, ethnicity, or other socially relevant groupings of the patients was made accessible to the researchers.
Population characteristics	Blood samples were collected from participants undergoing coronary artery bypass graft or elective cardiac valve replacement surgery.<br>Participants in AGI<2 group: BMI 22.2±1.2; median age/range: 53 (39 - 56) years; male:female ratio: 3:8 (0.38).<br>Participants in AGI ≥ 2 group: BMI 24.2 ± 0.6; median age/range: 59 (55 - 70) years; male:female ratio: 14:25 (0.56)
Recruitment	We used cardiac surgery as the intestinal I/R model for further analysis. We selected patients undergoing the same type ofoperation (including coronary artery bypass grafting or aortic valve replacement) while excluding patients who already hadgastrointestinal diseases before surgery. Patients were consecutively recruited and all participants were aged between 18and 75 years. No self-selection bias was anticipated.
Ethics oversight	All protocols used were approved by the Ethical Committee of the Nanfang Hospital, Southern Medical University (approval number: NFEC-202009-k2-01), and informed consent was obtained from all patients.

Note that full information on the approval of the study protocol must also be provided in the manuscript.

Field-specific reporting

Please select the one below that is the best fit for your research. If you are not sure, read the appropriate sections before making your selection.

☒ Life sciences ☐ Behavioural & social sciences ☐ Ecological, evolutionary & environmental sciences

For a reference copy of the document with all sections, see nature.com/documents/nr-reporting-summary-flat.pdf

Life sciences study design

All studies must disclose on these points even when the disclosure is negative.

Sample size	We have adopted at least n=3 biological replicates to calculate the statistical value of each analysis based on the previous publications in the filed (Cell.2020;180(6):1198-1211.e19.).
Data exclusions	No data were excluded from analysis.
Replication	All experiments were performed at least there times independent biological replicates in this paper. All attempts at replication were successful.
Randomization	Samples were randomly assigned to the control group or the experimental group in vivo and in vitro studies of this paper.
Blinding	The investigators were blinded to group allocation during all data collection and analysis process.

Reporting for specific materials, systems and methods

We require information from authors about some types of materials, experimental systems and methods used in many studies. Here, indicate whether each material, system or method listed is relevant to your study. If you are not sure if a list item applies to your research, read the appropriate section before selecting a response.

Materials & experimental systems

n/a	Involved in the study
☐	☒ Antibodies
☒	☐ Eukaryotic cell lines
☒	☐ Palaeontology and archaeology
☐	☒ Animals and other organisms
☒	☐ Clinical data
☐	☒ Dual use research of concern
☒	☐ Plants

Methods

n/a	Involved in the study
☒	☐ ChIP-seq
☐	☒ Flow cytometry
☒	☐ MRI-based neuroimaging

Antibodies

Antibodies used

Anti-Rabbit hnRNPA1 (Cat # YT2192; ImmunoWay, Plano, USA, WB/IF) at 1:300 of dilution.
Anti-Rabbit GPRC5A(Cat #YT3995; ImmunoWay, Plano, USA, WB/IF) at 1:500 of dilution.
Anti-Rabbit CD9 (Cat #ab92726; Abcam, WB) at 1:400 of dilution.
Anti-Rabbit CD63 (Cat #ab217345; Abcam, WB) at 1:400 of dilution.
Anti-Rabbit CD81 (Cat #10037;Cell Signaling Technology, WB) at 1:400 of dilution.
Anti-Rabbit Tsg101(Cat #ab125011; Abcam, WB) at 1:400 of dilution.
Anti-mouse Brdu (Cat #A1482; Abclonal, IHC/IF) at 1:600 of dilution.
Anti-Rabbit ZO-1 (Cat #ab221547; Abcam, IF) at 1:400 of dilution.
Anti-Rabbit occludin(Cat #ab216327; Abcam, IF) at 1:400 of dilution.
Anti-mouse Ki67 (Cat# ab279653; Abcam, IHC/IF) at 1:500 of dilution.
Anti-Rabbit OLFM4 (Cat#39141S; Cell Signaling Technology@ IF) at 1:400 of dilution.
Anti-Rabbit CyclinB1 (Cat # ab181593; Abcam, USA, WB) at 1:1000 of dilution.
Anti-Rabbit Myc (Cat # ab32072; Abcam, USA, WB) at 1:1000 of dilution.
Anti-Rabbit Bax (Cat # ab32503; Abcam, USA, WB) at 1:800 of dilution.
Anti-Rabbit c-Caspase3 (Cat # ab181593; Abcam, USA, WB) at 1:800 of dilution.
Anti-GAPDH Monoclonal antibody (10494-1-AP; Proteintech, Plano, USA, WB)
Chicken anti-GFP antibody (Abcam Cat #ab13970 , IF) at 1:400 of dilution.
Alexa Fluor 594-conjugated secondary antibody (711-585-152, Jackson Immunoresearch, IF) at 1:400 of dilution.
a Mo IgG/Alexa Fluor 594-conjugated secondary antibody (ZF-0513, ZSGB-BIO, IF) at 1:200 of dilution.

Validation

All of the antibodies used in this study were commercial and suitable for specific purposes. The antibodies were validated based on the information from the manufacturer's instructions and were supported by multiple publications. All primary antibodies for western blot are rabbit, primary antibodies for immunofluorescence and IHC are rabbit or mouse.

Anti-Rabbit hnRNPA1 (Cat # YT2192; ImmunoWay, Plano, USA). Reactivity: Human, Mouse, Rat. Application: WB;IHC;IF;ELISA.
Anti-Rabbit GPRC5A(Cat #ab188905; Abcam, USA). Reactivity: Mouse, Rat, Hamster, Cow, Dog, Monkey, Gorilla. Application: IHC-P.
Anti-Rabbit CD9 (Cat #ab92726; Abcam). Reactivity: Human, Mouse. Application: WB, ICC/IF, IHC-P, Flow Cyt, IP.
Anti-Rabbit CD63 (Cat #ab217345; Abcam). Reactivity: Human, Mouse, Rat. Application: WB, ICC/IF.
Anti-Rabbit CD81 (Cat #10037;Cell Signaling Technology). Reactivity: Human, Mouse, Rat. Application: WB, ELISA.
Anti-Rabbit Tsg101(Cat #ab125011; Abcam). Reactivity: Human, Mouse, Rat. Application: Flow Cyt (Intra), ICC/IF, IHC-P, WB.
Anti-mouse Brdu (Cat #A1482; Abclonal). Reactivity: Human, Mouse, Rat. Application: WB, ELISA.
Anti-Rabbit ZO-1 (Cat #ab221547; Abcam). Reactivity: Human, Mouse, Rat. Application: Flow Cyt (Intra), ICC/IF, IHC-P.
Anti-Rabbit occludin(Cat #ab216327; Abcam). Reactivity: Mouse, Rat, Dog, Human. Application: WB, IHC-P, ICC/IF, IP, Flow Cyt (Intra).
Anti-mouse Ki67 (Cat# ab279653; Abcam,). Reactivity: Human, Mouse, Rat. Application: Flow Cyt (Intra), IHC-P, ICC/IF.
Anti-Rabbit OLFM4 (Cat#39141S; Cell Signaling Technology@). Reactivity: Human, Mouse, Rat. Application: WB, ELISA.
Anti-Rabbit CyclinB1 (Cat # ab181593; Abcam, USA). Reactivity: Human, Mouse. Application: Flow Cyt (Intra), ICC/IF, IHC-P, WB.
Anti-Rabbit Myc (Cat # ab32072; Abcam, USA). Reactivity: Human, Mouse, Rat. Application: Flow Cyt (Intra), WB, ICC/IF, ChIP-sequencing, IHC-P, IP.
Anti-Rabbit Bax (Cat # ab32503; Abcam, USA). Reactivity: Human, Mouse, Rat. Application: IHC-P, WB, IP, Sandwich ELISA.
Anti-Rabbit c-Caspase3 (Cat # ab214430; Abcam, USA). Reactivity: Mouse. Application: WB.
Anti-GAPDH Monoclonal antibody (10494-1-AP; Proteintech, Plano, USA). Reactivity: Human, Mouse, Rat. Application: FC, IF, IHC, IP, WB, ELISA.
Chicken anti-GFP antibody (Abcam Cat #ab13970). Application: WB, ICC/IF.

Animals and other research organisms

Policy information about studies involving animals; ARRIVE guidelines recommended for reporting animal research, and Sex and Gender in Research

Laboratory animals	C57BL/6 male mice (6–8 weeks old) were obtained from the animal center of Southern Medical University (Guangzhou, China). All animals were housed in a specific pathogen-free environment with controlled temperature (20–22°C) and humidity (50–70%) , a 12/12 h light/dark cycle, and free access to food and water.
Wild animals	No wild animal has been used in this study.
Reporting on sex	All animals used in this study were male, no sex-based analysis was performed.
Field-collected samples	No field-collected samples were used in this study.
Ethics oversight	All experimental procedures involving animals were performed according to the National Institutes of Health guidelines and werereviewed and approved by the Ethics Committee of the Nanfang Hospital of Southern Medical University (approval number:NFYY-2019-0754).

Note that full information on the approval of the study protocol must also be provided in the manuscript.

Flow Cytometry

Plots

Confirm that:

- [x] The axis labels state the marker and fluorochrome used (e.g. CD4-FITC).
- [x] The axis scales are clearly visible. Include numbers along axes only for bottom left plot of group (a 'group' is an analysis of identical markers).
- [x] All plots are contour plots with outliers or pseudocolor plots.
- [x] A numerical value for number of cells or percentage (with statistics) is provided.

Methodology

Sample preparation	Sample preparation methods are detailed in the Methods section
Instrument	Flow cytometry was performed using FACS Calibur instruments (BD Biosciences, San Jose, CA, USA).
Software	All data were analyzed using FlowJo (Tree Star Inc.).
Cell population abundance	The analysis required a minimum of 5,000 cells.
Gating strategy	The positive cell population were isolated by specific antibody staining.

- [ ] Tick this box to confirm that a figure exemplifying the gating strategy is provided in the Supplementary Information.
